# Madmen and Their Friends

**Published:** 1892-08-27

**Authors:** 


### Madmen and their Friends.

A lunatic is not an easy person to handle, but sometimes, in the opinion of those gentlemen whom the public with charmingly discourteous inaccuracy generally style "mad doctors," the lunatic's friends are not much more easily managed. Of course every doctor suffers from his patients' friends and relatives. He asks for a history of the case he is called in to cure, and is rewarded with a long chapter of family biography, remarkable only for its irrelevance to the matter in hand. When the disease is insanity, the irrelevance is increased, and it is infinitely more irrepressible, for one must listen attentively on the bare chance that in the long meandering narrative some fact may be stated which has a diagnostic value, and will be a guide in treatment. But then what an intolerable deal of sack there is to this poor pennyworth of real information! There really is in many cases a literal and liberal supply of sack, or of its modern substitutes, beer and whiskey, to set the tale a-flowing. The relatives feel not only grieved but ashamed that one of the family should go out of his mind, and require to brace themselves with alcohol in order to undertake the task of depositing him in an asylum. Hence arise incoherence, manifestations of grief, and a general style of conduct eminently calculated to aggravate the patient's condition.

We may often add as one of the causes of the eccentric behaviour of relatives who bring patients to an asylum the consciousness of deception. They have not told the afflicted one of his destination. They merely suggested that it was a nice day for a drive, or something of the sort, and, keeping up the deception with one little falsehood after another, get him to the asylum, where without warning he finds himself landed, stranded, and deserted. No wonder that the excitable temperament of the patient who is thus deceived is roused to a nervous terror or delirious rage that makes the task of doctors and attendants doubly difficult. It is not always possible, indeed, to tell a patient that his disease renders it necessary for him to be put under restraint—though in many cases he knows his own weakness, and will submit readily enough—but at least he should know that he is going to change his abode and that he will be separated from his friends.

It is possible that the separation may be complete enough. The very state of nervous distress with which people hand over their friends to the authorities of an asylum makes them avoid returning there to see the patient. To do so implies the renewal of an unpleasant experience which many have not the courage to face. Dr. Elkins,* who speaks out of the fulness of his experience "concerning the kinsmen and friends of insane patients," says that, taking the average, husbands and wives are very prone to neglect the afflicted partner; while the most faithful visitors are mothers and maiden aunts. The unwearying love of the mother we understand and expect, but there is something very touching in the loyalty of the maiden aunt. She is a personage not too highly esteemed by the world at large; her little eccentricities of dress and demeanour are subjected to the keenest comment; her prejudices are mocked at by matron sisters every whit as full of caprice; nephews and nieces grumble that she does not share all their advanced ideas; unless she has wealth enough to make her will an object of interest she is generally neglected as being "a sour old maid." Solitude is her portion in life; but it is she who can find love to remember, and time and courage to visit, the kinsman who dwells in a mental isolation more lonely still.

It must be admitted, however, that visitors are not always beneficial to the patient. Well-meaning they may be, but injudicious they certainly are. They bring presents—sweetmeats to upset the digestion, alcohol to inflame the brain. One even brought a lunatic a razor—to shave his chin or cut his neighbour's throat with! Even when they do not thus do physical mischief, the visit of a hysterical relative to a melancholic patient is not a thing to be viewed with satisfaction. It must be remembered that the near relations of insane patients are often, in vulgar parlance, "tarred with the same stick." They may not be mad, but they are excitable, unreasonable, distinctly unwise. They tell the hapless dweller in the asylum of deaths in the family, of widows left destitute and orphans homeless, and take his ready tears as proof that "he understood quite well." Or they rouse him to anger by narrating how, in their opinion, Uncle John is mismanaging the family property, and would fain lure him into some demonstration against a relative who, in all probability, is honestly performing a thankless task. But even friends, about whose sanity there can be no manner of doubt, often behave in a way that could only be described as heartless, if it were not so often stupid. They seem to think that because a patient is mad on one point or another he has absolutely no knowledge of his native tongue, and talk about him in his presence in a way that can be excused only by the charitable assumption that they think the listener is deaf and a hopeless idiot to boot. They describe all his foolish acts, they speak of his possible death, and make suggestions for his funeral, in apparent forgetfulness of the fact that they are dealing with a human being of rather extreme nervous sensibility.

On the other hand, there is the visitor who forgets that a lunatic is mad at all, and swallows his every word with unreasoning credulity. Some people are so convinced that an asylum is an abode of horror that they actually invite the patient to complain of every restraint, and really believe, on a madman's word, that blows and starvation form more than half the treatment. They forget that when at home the patient made the very same complaints; and they forget still more completely that it was they themselves who put questions calculated to induce complaints. It is quite possible to say, "Are you sure they are kind to you?" or "Do you always get enough to eat, and whatever you want?" in such a tone as to get a negative reply from wiser people than the average inmate of an asylum. The mere suggestion that there may be neglect or unkindness sets one searching one's imagination for an example.

In fact, it is so extremely rare for visitors to an asylum to come with the same calm unprejudiced mind with which they would pay an ordinary visit, that it is no wonder that their conduct there is a little eccentric, so much so occasionally that the older patients take them for new cases. They may be distressed by the one or two signs of aberration which they see; but in general they are looking for even more, and in their habit of "talking down" to patients, who may, save on one point, be of distinctly acute intellect, they make themselves ridiculous and even offensive to their sensitive hearers. The cure for such follies is more frequent visits, a true familiarity with the asylum and its occupants. And those who are inclined to do this will invariably find that physicians and managers are pleased to welcome anything or anyone who can give brightness to the lives of those under their charge.

* Concerning the Kinsmen and Friends of Insane Patients: By Frank Ashby Elkins, M.B., Assistant Physician, Royal Edinburgh Asylum.

---

Advertisement occasionally brings an unexpected and unwelcome reward. A quack in the States advertised that he had treated numerous cases of diphtheria with unfailing success. In reply to this advertisement, he was promptly arrested for having failed to report the cases to the Board of Health.

